# Comprehensive Telestroke Network to Optimize Health Care Delivery for Cerebrovascular Diseases: Algorithm Development

**DOI:** 10.2196/18058

**Published:** 2020-07-27

**Authors:** Hernán Bayona, Brenda Ropero, Antonio José Salazar, Juan Camilo Pérez, Manuel Felipe Granja, Carlos Fernando Martínez, Juan Nicolás Useche

**Affiliations:** 1 Primary Stroke Center Neurology Department University Hospital Fundación Santa Fe de Bogotá Bogotá DC Colombia; 2 College of Medicine University of Los Andes Bogotá DC Colombia; 3 Department of Diagnostic Imaging University Hospital Fundación Santa Fe de Bogotá Bogotá DC Colombia; 4 Electrophysiology and Telemedicine Laboratory University of Los Andes Bogotá DC Colombia; 5 Lyerly Neurosurgery Baptist Health Jacksonville, FL United States

**Keywords:** stroke, telestroke, cerebrovascular disease, software, algorithms, emergency medicine, clinical pathways

## Abstract

**Background:**

Health care delivery for cerebrovascular diseases is a complex process, which may be improved using telestroke networks.

**Objective:**

The purpose of this work was to establish and implement a protocol for the management of patients with acute stroke symptoms according to the available treatment alternatives at the initial point of care and the transfer possibilities.

**Methods:**

The review board of our institutions approved this work. The protocol was based on the latest guidelines of the American Heart Association and American Stroke Association. Stroke care requires human and technological resources, which may differ according to the patient’s point of entry into the health care system. Three health care settings were identified to define the appropriate protocols: primary health care setting, intermediate health care setting, and advanced health care setting.

**Results:**

A user-friendly web-based telestroke solution was developed. The predictors, scales, and scores implemented in this system allowed the assessment of the vascular insult severity and neurological status of the patient. The total number of possible pathways implemented was as follows: 10 in the primary health care setting, 39 in the intermediate health care setting, and 1162 in the advanced health care setting.

**Conclusions:**

The developed comprehensive telestroke platform is the first stage in optimizing health care delivery for patients with stroke symptoms, regardless of the entry point into the emergency network, in both urban and rural regions. This system supports health care personnel by providing adequate inpatient stroke care and facilitating the prompt transfer of patients to a more appropriate health care setting if necessary, especially for patients with acute ischemic stroke within the therapeutic window who are candidates for reperfusion therapies, ultimately contributing to mitigating the mortality and morbidity associated with stroke.

## Introduction

Stroke is a major source of disability and death in both developed and developing countries [1-4]. The adequate delivery of care for patients with acute stroke symptoms requires the expertise of neurologists and radiologists for timely diagnosis and treatment. Whereas hemorrhagic stroke often requires urgent surgical intervention, ischemic stroke is managed with reperfusion therapies such as thrombolysis with intravenous recombinant tissue plasminogen activator (IV rtPA) as well as early endovascular thrombectomy in the case of large vessel occlusions. These approaches have significantly improved the long-term outcomes of patients with ischemic stroke [5]. Nevertheless, patients frequently do not receive the appropriate treatment either due to the lack of available specialists to perform appropriate clinical assessments or the long distances and prolonged transfer times to stroke care centers [5]. This situation can also occur for patients located in urban areas due to delays in the referral process to health care facilities with the required stroke handling capabilities.

We have developed several strategies to improve the quality of care and speed up the transfer of patients with acute stroke symptoms from urban and rural areas to our hospital, a certified primary stroke center with thrombectomy capabilities. Our experience has shown that, by necessity, robust stroke systems should be able to assist health care providers in real-time scenarios, thus resulting in adequate transfer processes between any level of complexity in a specific health care setting.

The purpose of this work was to establish a protocol for the management of patients with acute stroke symptoms according to the available treatment alternatives at the initial point of care. This protocol was implemented as a web-based telestroke solution and is based on the guidelines from the American Heart Association and American Stroke Association (AHA/ASA) [6] for any patient with acute stroke symptoms (ie, hemorrhagic stroke, acute or chronic ischemic stroke, transient ischemic attack [TIA], stroke mimics, and large vessel occlusions). The authors considered that a detailed description of the workflow protocols, pathways, and clinical and radiological scales used in the design of our telestroke network will help other maturing countries in the development of early-stage health systems assisting patients with acute stroke symptoms.

## Methods

The work presented here is part of a larger project with the following objectives: evaluation of mobile systems for head computed tomography (CT) interpretation in acute stroke patients, evaluation of the quality of stroke care in our country from a public health standpoint, and development of a telestroke network system (the subject of this article). This initiative was approved by the Institutional Review Board of our hospital and university.

The latest diagnostic and therapeutic recommendation guidelines from the American Heart Association [6], in addition to several predictors, scales, and scores that allow the assessment of the vascular insult severity and neurological status of the patient (Table 1), were evaluated in order to define the protocols of this system.

Stroke care requires human resources (eg, neurologists, radiologists, or neuroradiologists) to evaluate the risk and eligibility of patients to receive reperfusion therapies (intravenous thrombolysis or endovascular thrombectomy) and to perform invasive treatments when indicated. In addition, technological resources, such as CT, computed tomography angiography (CTA), magnetic resonance imaging (MRI), magnetic resonance angiography (MRA), and the necessary medical supplies and equipment, are needed to administer reperfusion therapies. Different human and technological resources may be available according to a patient’s point of entry into the health care system. Therefore, several possible health care settings were evaluated to define the protocols and algorithms of this system. The algorithms for the clinical workflow of the three health care settings were defined and reviewed by a group of experts in our hospital: a stroke neurologist, a general neurologist, a neuroradiologist, and two physicians from our stroke center.

**Table 1:** Neurological and radiological scales used in the stroke treatment processes.

Matrix or scale	Description
Glasgow Coma Scale [7,8]	Assesses the level of consciousness
National Institutes of Health Stroke Scale (NIHSS) [9-12]	Quantifies the clinical severity of ischemic stroke
Posterior circulation predictor [13]	Predicts posterior circulation involvement
ABCD2 score [14,15]	Predicts subsequent risk of stroke in patients with TIA^a^ diagnosed by emergency physicians
Field Assessment Stroke Triage for Emergency Destination (FAST-ED) [16]	Determines the probability of large-vessel occlusion
Intracerebral hemorrhage (ICH) score [17-19]	Grades early hemorrhage growth in patients with intracerebral hemorrhage
Fisher scale [20]	Grades the severity of the subarachnoid hemorrhage
Modified World Federation of Neurosurgical Societies (WFNS) [21]	Grades the severity of subarachnoid hemorrhage based on the Glasgow Coma Scale
BE-FAST score [22]	Evaluate potential stroke before physician evaluation to activate the stroke code
Alberta Stroke Program Early CT Score (ASPECTS) [23]	Can estimate the infarction size of the middle cerebral artery territory
Reperfusion Therapy Risk Mitigation [24]	Assesses the absolute and relative risks of thrombolysis or thrombectomy
Thrombolysis in Cerebral Infarction (TICI) score [25]	Addresses the extent of tissue reperfusion

^a^TIA: transient ischemic attack.

## Results

**Figure 1:**
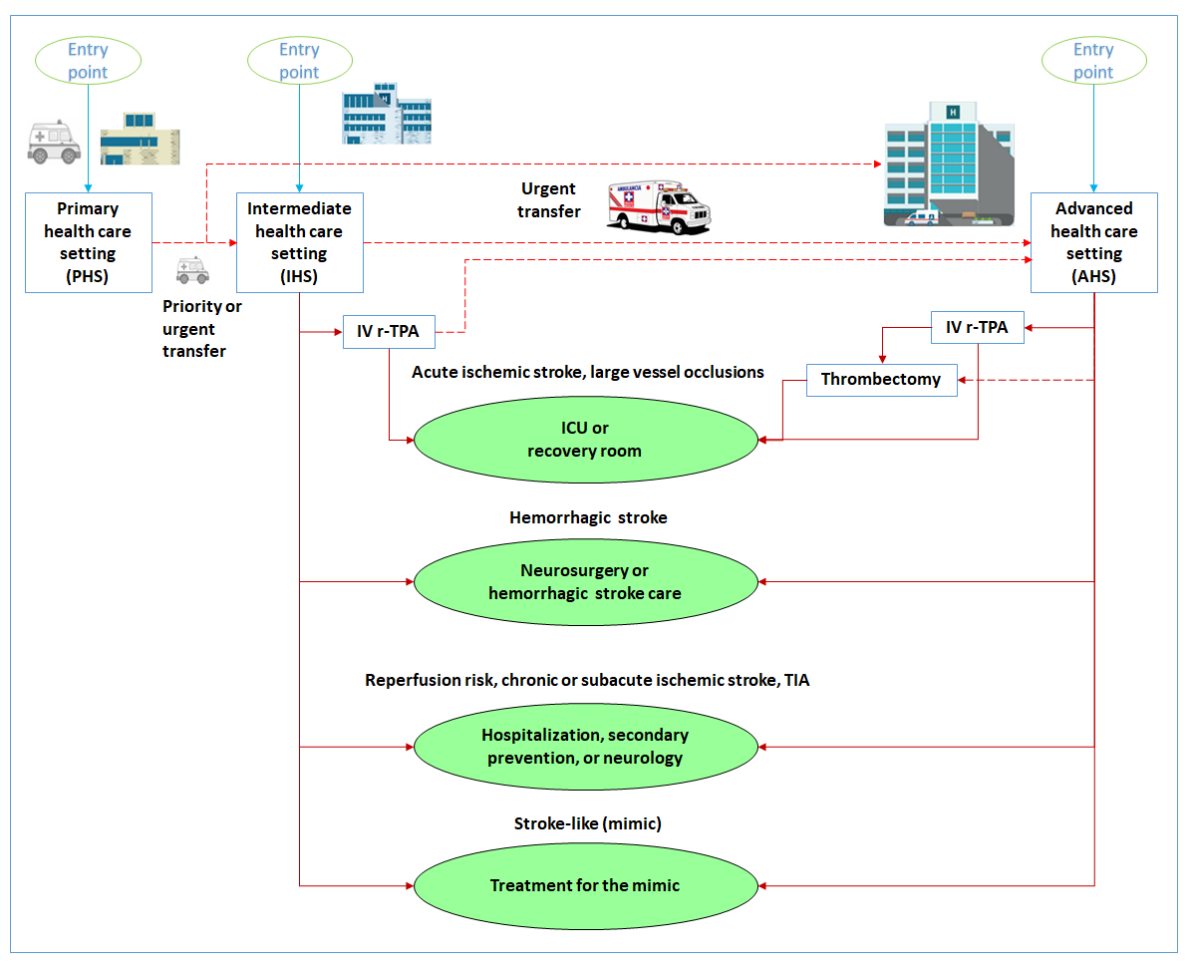
Interaction between the three health care settings and final possible diagnosis and referrals. ICU: intensive care unit; IV rtPA: intravenous recombinant tissue plasminogen activator; TIA: transient ischemic attack.

The following health care settings were identified: primary health care setting, intermediate health care setting, and advanced health care setting. The interaction between these three settings is shown in Figure 1, which also shows the optimal health care settings according to a specific patient diagnosis (eg, intensive care unit [ICU], recovery room, facility with neurology or neurosurgery capabilities, regular hospitalization, or ambulatory care). IV rtPA administration can be provided in the intermediate health care setting or advanced health care setting, while thrombectomy is performed only in the advanced health care setting. In both cases, a judicious risk assessment is needed before the administration of any treatment modality [6].

In all settings, the first step is the acquisition of demographic data followed by a clinical background update, blood glucose registry, physical exam, assessment of the level of consciousness using the Glasgow Coma Scale [7], and assessment of the clinical severity of the ischemic stroke using the National Institutes of Health Stroke Scale (NIHSS) [9]. The next step is to determine the onset time to calculate the therapeutic window time (ie, the time between neurological symptom onset and patient arrival to the emergency room). In some cases, the event may be classified as wake-up or unwitnessed stroke. The therapeutic window time may be “within window” (<6 hours) or “out of window” with two possible ranges (6-24 hours or >24 hours) that determine the differences in patient management. The combination of the NIHSS and Glasgow scores as well as the time from onset to care delivery are critical breakpoints for the decision-making process performed at each of the three health care settings.

### Health Care Settings

**Figure 2:**
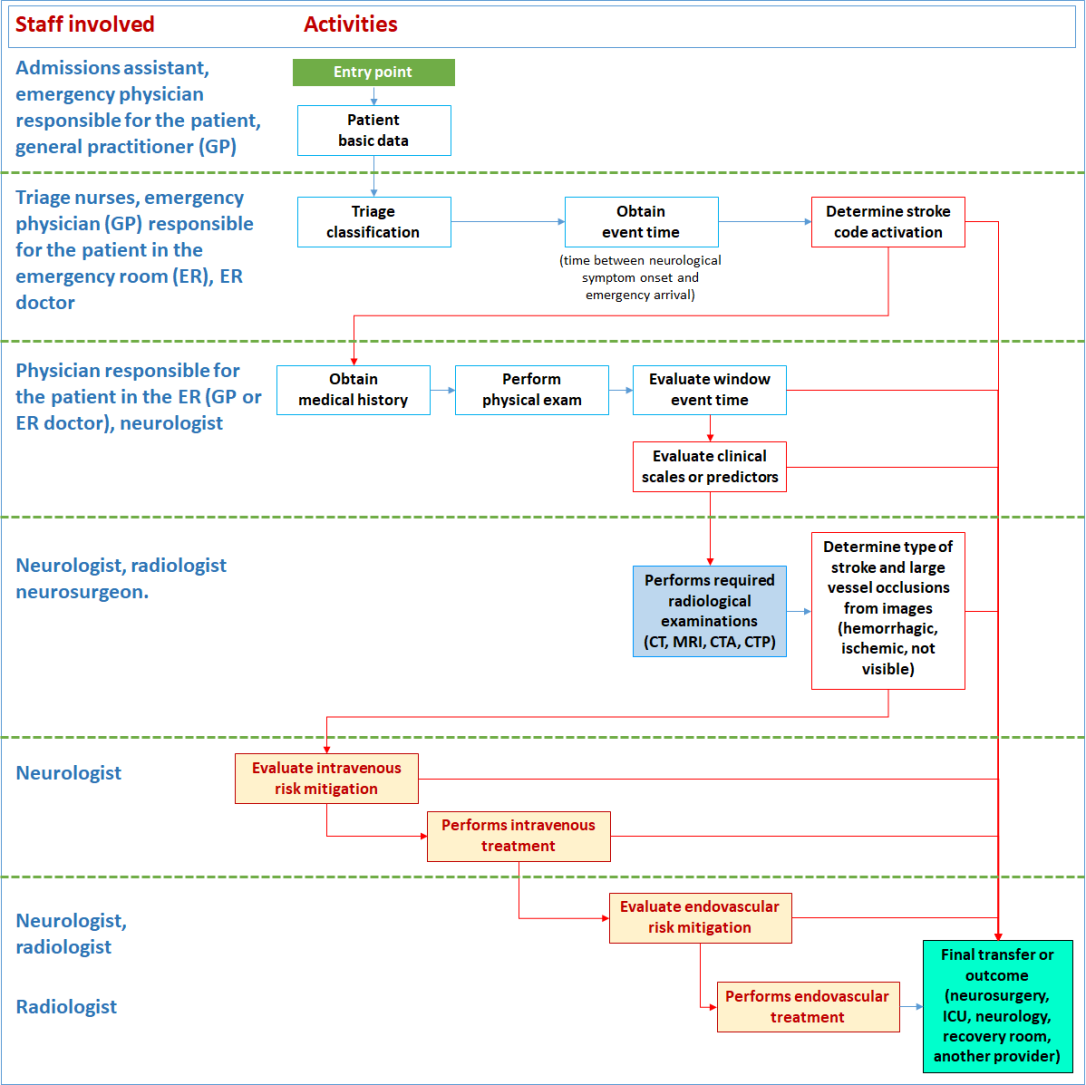
General simplified workflow for the three health care settings. CT: computed tomography; CTA: computed tomography angiography; CTP: computed tomography perfusion; ICU: intensive care unit; MRI: magnetic resonance imaging.

The general diagnostic and treatments steps, within each health care setting, are presented in a simplified workflow shown in Figure 2.

#### Primary Health Care Setting

**Figure 3:**
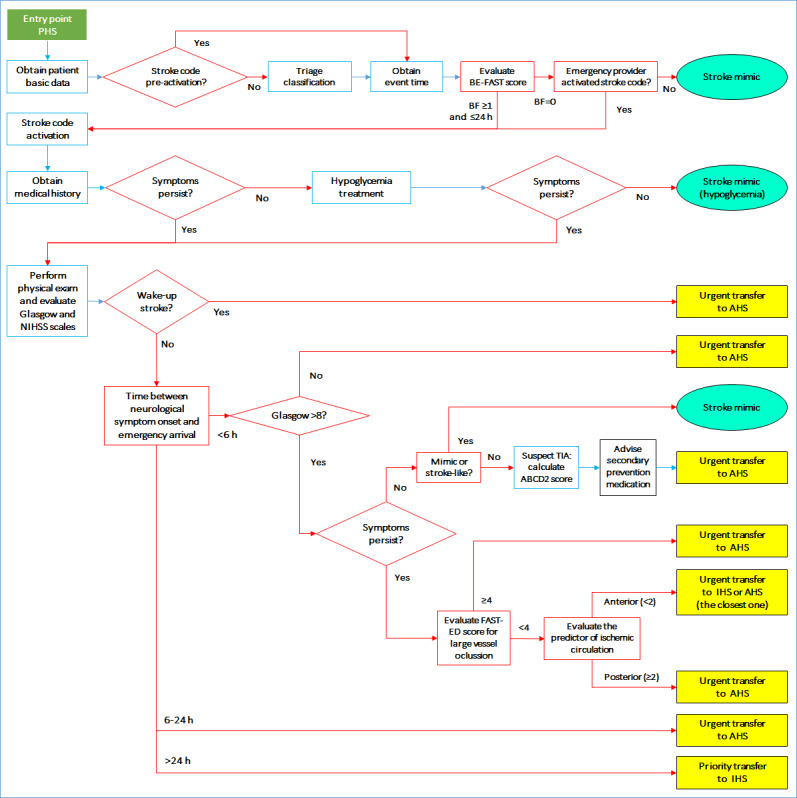
Workflow for the primary health care setting (PHS). AHS: advanced health care setting; Glasgow: Glasgow Coma Scale; FAST-ED: Field Assessment Stroke Triage for Emergency Destination; IHS: intermediate health care setting; NIHSS: National Institutes of Health Stroke Scale; TIA: transient ischemic attack.

In this setting, diagnostic tools are limited to the physical exam performed by a primary care physician as well as basic blood tests (eg, blood glucose). This setting may include ambulances, which may be a possible entry point to the health care system. The purpose of this setting is to provide an initial clinical assessment and determine the patient’s transfers to a health care center with reperfusion capabilities, according to possible TIA, large vessel occlusion, or ischemic stroke in the anterior or posterior circulation. In this scenario, there are neither imaging facilities (CT or MRI) nor specialized health care personnel. Therefore, different clinical scales are used to assess the patient’s risk at multiple levels and to predict final patient outcomes. For example, patients with possible compromised posterior circulation will be transferred to an advanced health care setting, while those with anterior circulation may be transferred to any intermediate or advanced health care site; this estimation is achieved using ischemic stroke circulation predictors [13,26]. The ABCD2 score [14] is a powerful tool that predicts the subsequent risk of stroke after a TIA, and the Field Assessment Stroke Triage for Emergency Destination score is used to determine the probability of a large-vessel occlusion [16]. The detailed workflow for this setting is shown in Figure 3.

#### Intermediate Health Care Setting

At this level of health care facility, head CT must be available to detect hemorrhagic or ischemic stroke; intravenous thrombolysis capabilities are also required at this level.

**Figure 4:**
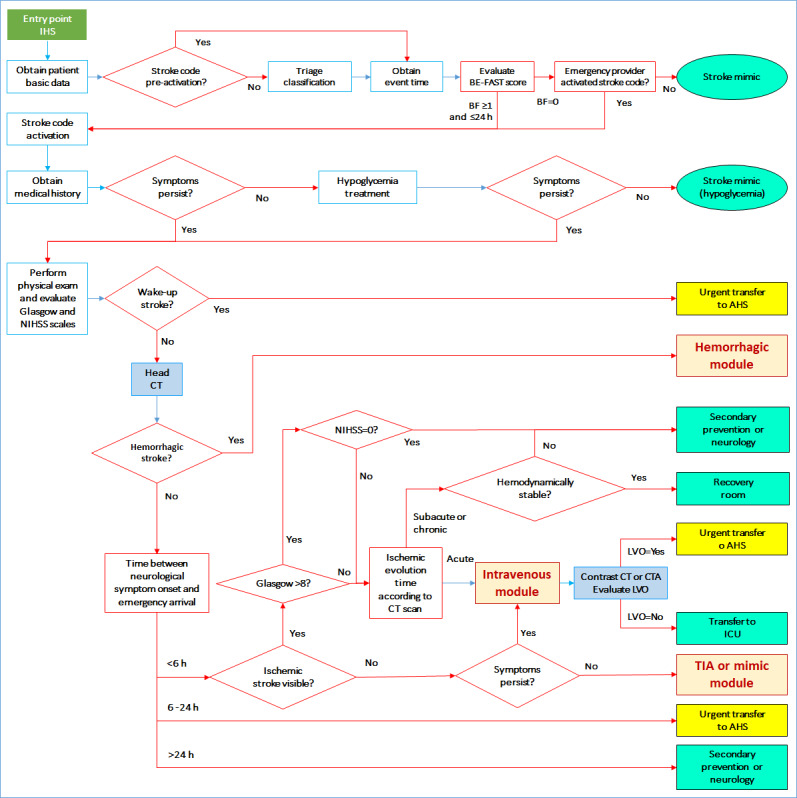
Workflow for the intermediate health care setting (IHS). AHS: advanced health care setting; CT: computed tomography; CTA: computed tomography angiography; Glasgow: Glasgow Coma Scale; ICU: intensive care unit; LVO: large vessel occlusion; NIHSS: National Institutes of Health Stroke Scale; TIA: transient ischemic attack.

To diagnose a potential large vessel occlusion and determine if further transfer to the advanced health care setting is necessary, CTA must be available (or contrast head CT if CTA is not available). At the intermediate health care setting, both neurologists and radiologists may be available, but not fulltime. This setting works as a mothership for urgent and priority transfers from the primary health care setting. Possible outcomes include priority transfer to an advanced health care setting for thrombectomy purposes, emergent assessment by the neurology or neurosurgery teams, IV r-TPA administration, or ambulatory care. In this setting, if a patient is eligible for IV r-TPA administration, this is done in situ, to enable early treatment, even if the patient will be transferred to the advanced health care setting. The detailed workflow for this setting is shown in Figure 4.

#### Advanced Health Care Setting

**Figure 5:**
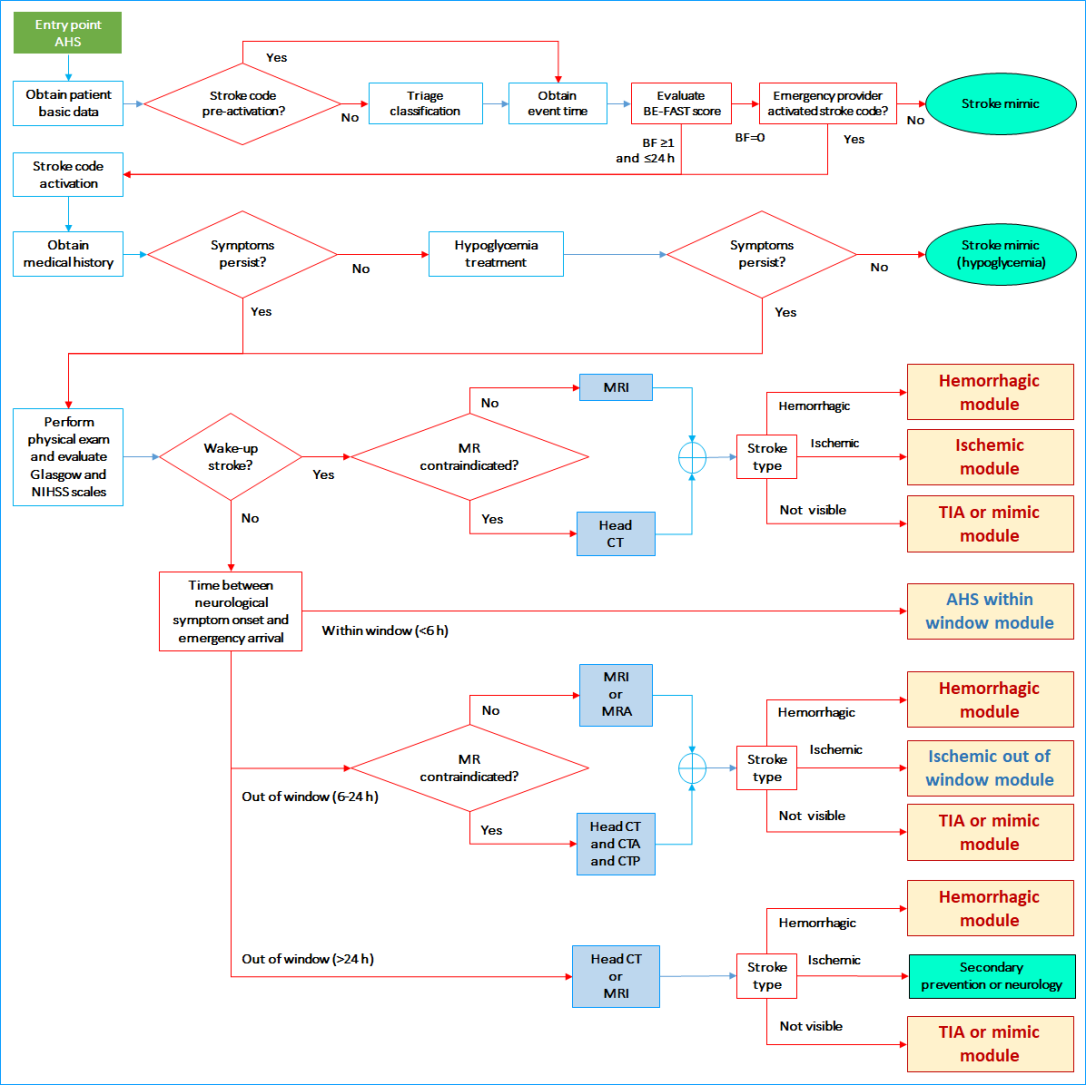
Workflow for the advanced health care setting (AHS). CT: computed tomography; CTA: computed tomography angiography; CTP: computed tomography perfusion; Glasgow: Glasgow Coma Scale; MR: magnetic resonance; MRA: magnetic resonance angiography; MRI: magnetic resonance imaging; NIHSS: National Institutes of Health Stroke Scale; TIA: transient ischemic attack.

**Figure 6:**
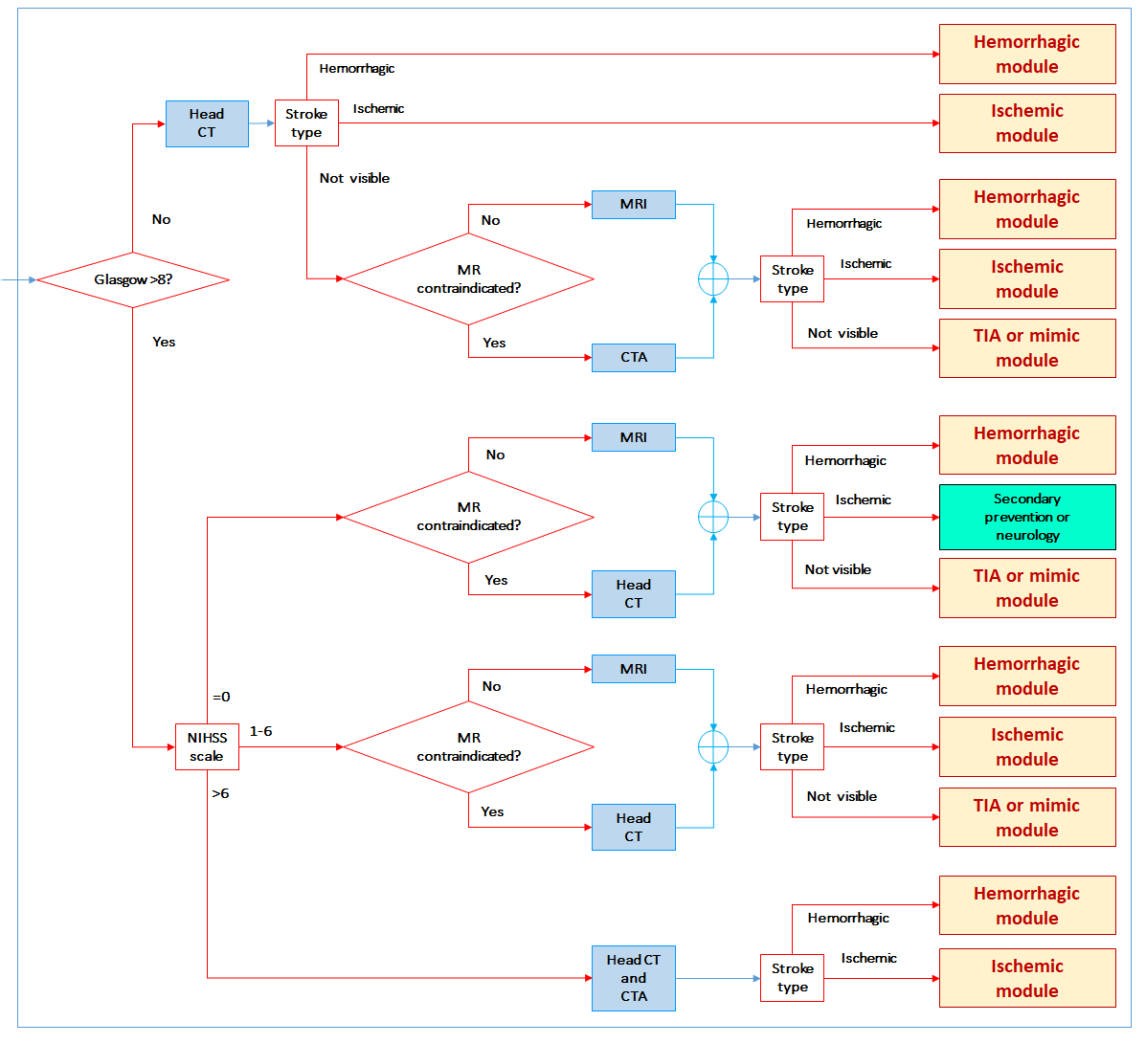
Within window module for the advanced health care setting. CT: computed tomography; CTA: computed tomography angiography; Glasgow: Glasgow Coma Scale; MR: magnetic resonance; MRI: magnetic resonance imaging; NIHSS: National Institutes of Health Stroke Scale; TIA: transient ischemic attack.

**Figure 7:**
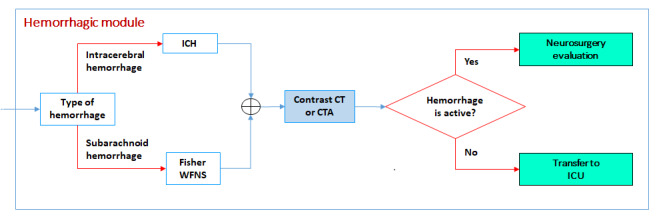
Hemorrhagic module. CT: computed tomography; CTA: computed tomography angiography; ICH: intracerebral hemorrhage score; ICU: intensive care unit; WFNS: Modified World Federation of Neurosurgical Societies.

**Figure 8:**
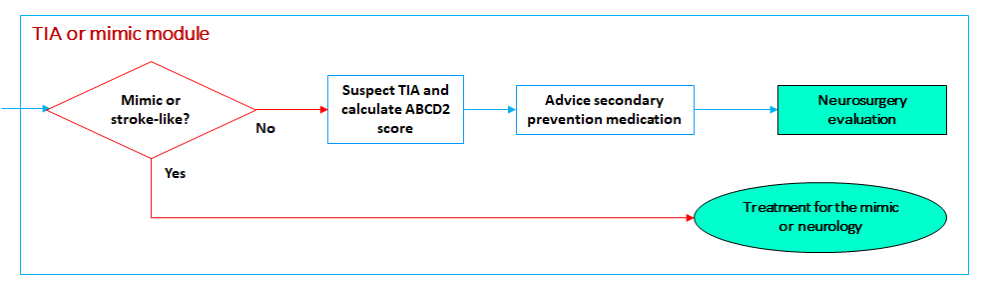
Transient ischemic attack (TIA) or mimic module.

**Figure 9:**
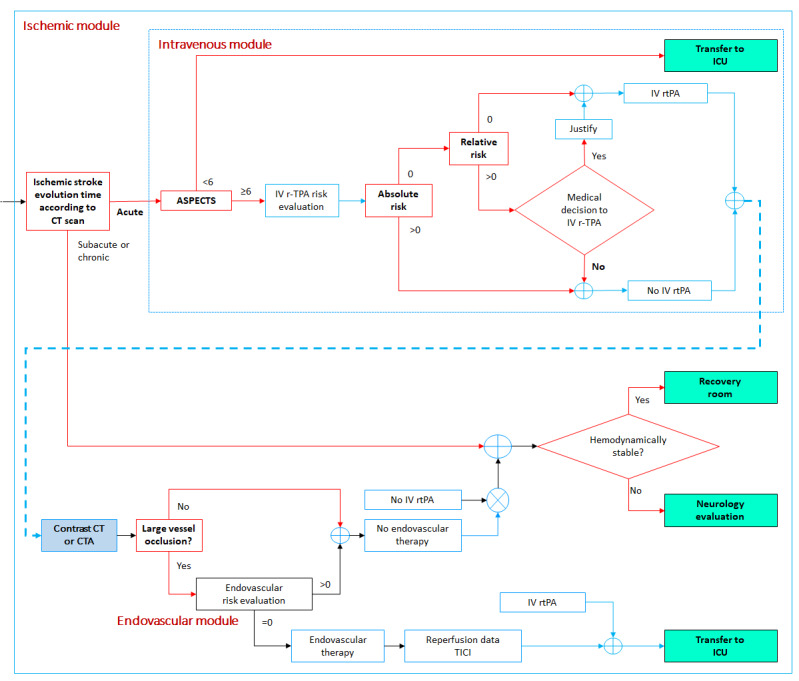
Ischemic module. ASPECTS: Alberta Stroke Program Early Computed Tomography Scan; CT: computed tomography; CTA: computed tomography angiography; ICU: intensive care unit; IV rtPA: intravenous recombinant tissue plasminogen activator; TICI: Thrombolysis in Cerebral Infarction scale.

**Figure 10:**
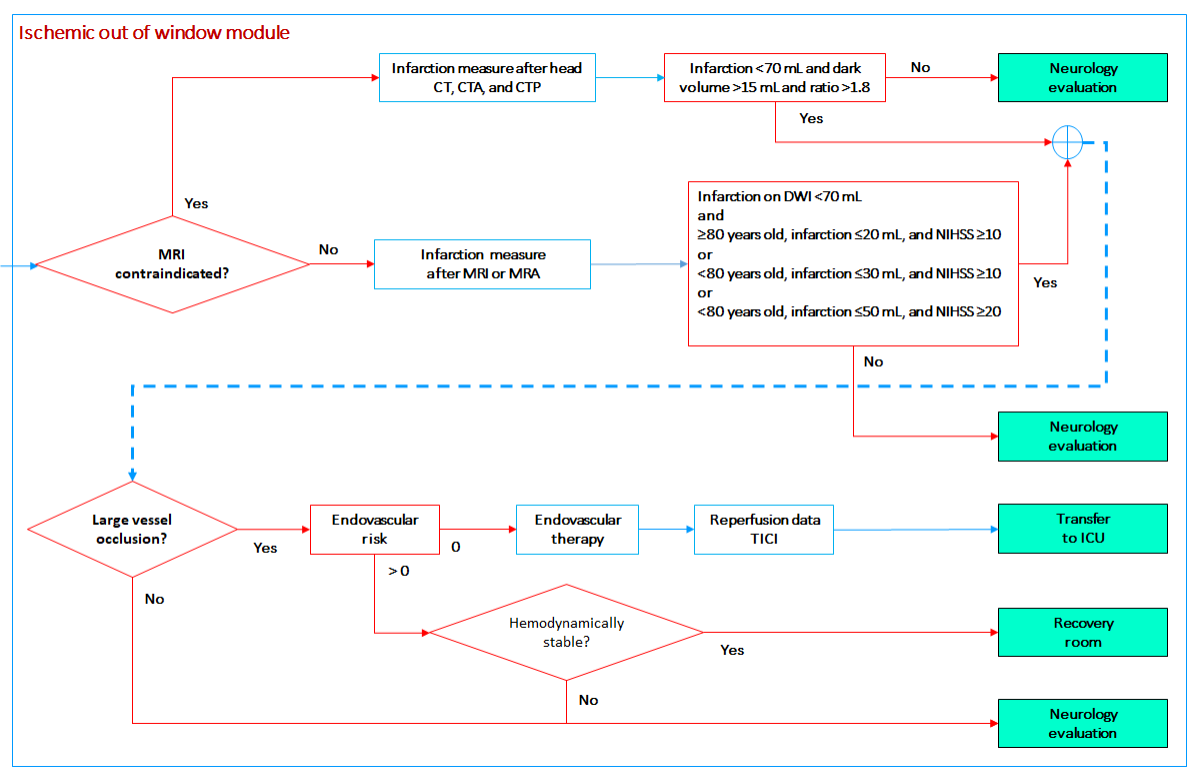
Ischemic stroke out of window module. CT: computed tomography; CTA: computed tomography angiography; CTP: computed tomography perfusion; DWI: diffusion-weighted magnetic resonance imaging; ICU: intensive care unit; MRA: magnetic resonance angiography; MRI: magnetic resonance imaging; NIHSS: National Institutes of Health Stroke Scale; TICI: Thrombolysis in Cerebral Infarction scale.

In this health care setting, specialized human and technological resources, such as stroke neurologists, neuroradiologists, CT, CTA, MRI, MRA, and the capacity for thrombolysis and mechanical thrombectomy, are available fulltime. Therefore, interfacility transfers are not necessary. In this setting, CT perfusion images are required for patients with wake-up stroke for which MRI is contraindicated. This setting receives transfers from primary health care settings and intermediate health care settings. The possible outcomes are shown in Figure 5. The workflow in the advanced health care setting for patients within the window for thrombolysis (ie, <6 hours) is shown in Figure 6. To simplify the workflow figures and render each figure on a single page, several common procedures in the intermediate and advanced health care settings were arranged in modules presented in Figures 7-10 (ie, hemorrhagic module, TIA or mimic module, intravenous thrombolysis module, endovascular treatment module, and ischemic stroke out of window module).

### Common Modules

#### Hemorrhagic Module

If the imaging examination shows a hemorrhagic stroke, the Fisher scale [20] and modified World Federation of Neurosurgical Societies scale [21] are used to evaluate the severity of the subarachnoid hemorrhage. To predict mortality in patients with intracerebral hemorrhage, the intracerebral hemorrhage score [17-19] is used. CT or CTA may reveal active intracranial bleeding; in this case, the patient is referred to the neurosurgery team for urgent care. Otherwise, the patient can be stabilized and treated in the ICU (Figure 7).

#### TIA or Mimic Module

If the symptoms are gone or are not consistent with a vascular territory and imaging examination reveals neither a hemorrhagic stroke nor an ischemic stroke, there are two possibilities: the patient is presenting with a stroke mimic or having a TIA. In the later, the ABCD2 score [14] is calculated to evaluate the actual stroke risk. Secondary prevention using statins and antiaggregant therapy is initiated, and the patient is discharged for neurologic outpatient care (Figure 8).

#### Ischemic Module

This module consists of two submodules: intravenous thrombolysis module for IV rtPA administration and endovascular treatment module, to evaluate thrombectomy treatment (Figure 9).

#### Intravenous Thrombolysis Module

If the initial imaging examination reveals an ischemic stroke, an imaging vascular evaluation of the anterior and posterior circulation is performed according to the onset time of the ischemic insult (acute, subacute, or chronic). Patients with subacute or chronic lesions are referred to the recovery room if hemodynamically stable; otherwise, they are hospitalized for neurology assessment. For patients with acute lesions in the middle cerebral artery territory, the Alberta Stroke Program Early CT Scan (ASPECTS) is calculated; patients with ASPECTS <6 are referred to the ICU, whereas patients with ASPECTS ≥6 are evaluated in terms of the absolute and relative contraindications for IV rtPA administration (Table 2) [24]. Next, if there is no risk or only a relative risk after judicious medical assessment, intravenous thrombolysis is performed. At the same time, the patient is evaluated for the presence of large vessel occlusions and possible thrombectomy, as indicated in the endovascular treatment module (Figure 9).

**Table 2:** Risk mitigation matrices for reperfusion therapies.

Risk mitigation treatment		Contraindications
**Intravenous thrombolysis – absolute criteria**		
	Clinical history	Previous ischemic stroke within 3 months; previous intracranial hemorrhage (excluded hemorrhagic transformation of a stroke); intra-axial neoplasm at this time; craniocerebral trauma or spinal within the inpatient acute period or within the 3 previous months; intracranial or spinal surgery in the last 3 months; infectious endocarditis or actual aortic dissection; extra-axial neoplasia, arteriovenous malformation, or aneurysm not excluded; arterial puncture in noncompressible location not in the last 7 days
	Incoming clinical parameters	Suggestive symptoms of subarachnoid hemorrhage; blood pressure ≥185/110 mm Hg, despite management according to the protocol; blood glucose levels <50 mg/dL; active internal bleeding or active hemorrhagic diathesis
	Hematologic	Platelet count <100,000/mm^3^; INR^a^ >1.7 and PT^b^ >15 seconds or PTT^c^ >40 seconds; heparin (last 48 hours with an abnormal PTT), heparin of low molecular weight in therapeutic doses (last 48 hours); inhibitors of thrombin/Xa factor in the last 48 hours
	Diagnostic imaging	Evidence of acute intracranial hemorrhage, infarction size of the middle cerebral artery territory hemisphere ≥1/3 (ie, ASPECTS^d^ <6)
	Special cases	Start of pregnancy until 14 days postpartum; window 3-4.5 hours, ≥80 years old, diabetes, previous stroke, use of oral anticoagulant
**Intravenous thrombolysis – relative criteria**		
	Clinical history	Surgery or major trauma in the last 14 days; gastrointestinal tract or urinary tract hemorrhage in the last 21 days; acute myocardial infarction, especially with segment ST elevation or pericarditis in the last 3 months
	Incoming clinic parameters	NIHSS^e^ <4 or NIHSS ≥25
**Thrombectomy – absolute criteria**		
	Physical exam	NIHSS >29
	Hematologic	INR >3, PTT >2, thrombocytopenia <30,000/mm^3^
	Diagnostic imaging	ASPECTS <6, infarction size (on DWI^f^) >70 mL, active hemorrhage

^a^INR: international normalized ratio.

^b^PT: prothrombin time.

^c^PTT: partial thromboplastin time.

^d^ASPECTS: Alberta Stroke Program Early Computed Tomography Scan.

^e^NIHSS: National Institutes of Health Stroke Scale.

^f^DWI: diffusion-weighted magnetic resonance imaging.

#### Endovascular Treatment Module

Large vessel occlusions are evaluated using contrast CT or CTA. If there are no occlusions, the patient is referred to the recovery room if hemodynamically stable; otherwise, they are hospitalized for continuous neurologic assessment. If a large vessel occlusion is confirmed, a comprehensive risk evaluation should be performed before thrombectomy [24]. Then, if no risks are identified, thrombectomy should be performed as soon as possible. After thrombectomy is performed, the degree of reperfusion is measured by means of the Thrombolysis in Cerebral Infarction score [25], and the patient is referred to the ICU (Figure 9).

#### Ischemic Stroke Out of Window Module

Patients arriving to an advanced health care setting after a wake-up stroke, unwitnessed stroke, or “out of window” stroke with symptom onset 6-24 hours before first medical contact may benefit from reperfusion therapies only if certain conditions are met (Figure 10). These conditions rely on the infarct volume as quantified by diffusion-weighted MRI or CT perfusion (if MRI is contraindicated), patient age, and stroke severity (NIHSS score).

### Design of Algorithms

The three algorithms work as a handy framework for the most critical steps in the care of patients with ischemic or hemorrhagic stroke. These algorithms were based on decision trees that represent the clinical requirements and specifications of the system and consist of checklists and questionnaires [27] evaluating common physiological variables, the patient’s clinical background, different predictors and scales, and specific laboratory tests according to each algorithm stage. The responses to these questionnaires determine the next step to be performed.

The predictors, scales, and scores implemented in this system allow the assessment of the vascular insult severity and neurological status of the patient. Other factors that determine patient management, either in situ or in a distant health care setting, are shown in Table 1. The clinical background, physical exam, stroke severity, and radiological findings were stored in basic modules within the clinical algorithms. These modules, as implemented in our telestroke system, are shown in Table 3. The software outputs corresponding to specific diagnoses, clinical scenarios, and transfer decisions are shown in Table 4. The checklists for risk assessment before the administration of reperfusion therapies [24] are detailed in Table 2.

**Table 3:** Input information modules implemented in the telestroke system.

Input data		Examples of possible data
Identification data		Name, age, sex, identification number, date of birth
**Triage module**		
	Short physical exam	Heart rate, blood pressure, respiratory rate, weight
	Triage level	1 or 2
	Incoming patient medium	By their own means, referral from another service, ambulance transfer
	Cincinnati scale	0-3
	Stroke code activation?	Yes/no
**Clinical history module**		
	Neurological exam	Dysarthria, hemiparesis, hemiplegia, agnosia, aphasia, dysphagia, paresthesia, mutism, headache, delirium, loss of touch, facial weakness sparing the forehead
	Past relevant illnesses	Previous stroke, diabetes mellitus, dyslipidemia, coronary disease, chronic kidney disease, atrial fibrillation, high blood pressure, sleep apnea or hypopnea syndrome, smoking, thrombophilia
	Relevant findings	Patient found unconscious; patient woke with loss of sensation in the one side of her body
	Use of anticoagulant?	Yes/no; if yes, which one: apixaban, dabigatran, rivaroxaban, enoxaparin, warfarin, edoxaban
	Laboratory	Blood glucose levels, clotting times (PT^a^ and aPTT^b^), platelet count, INR^c^, blood count
**Physical exam module**		
	Cardiopulmonary resuscitation?	Yes/no
	Intubation?	Yes/no
	Glasgow Coma Scale	3-15
	NIHSS^d^	0-37
**Event time**		
	Time of onset of symptoms	Observed date and time or wake-up or unwitnessed stroke
	Time of arrival at emergency door	YYYY/MM/DD hh:mm
	Evolution time (hours)	Calculated from the previous times or typed directly
Therapeutic window type		Within therapeutic window (<6 hours), out of therapeutic window (>6 hours) or (6-24 hours; >24 hours), or wake-up or unwitnessed stroke
ABCD2 score; stroke risk after a TIA^e^		0-7; percentage (%) at 2, 7, and 90 days
Imaging evaluation (CT^f^, CT perfusion, CTA^g^, MRI^h^)		Compromised cerebral territory, ischemic event dating (acute, subacute, chronic), ASPECTS^i^, large vessel occlusions, or infarct volume, dark volume
Risk evaluation^j^		Absolute and relative evaluation for thrombolysis, risk evaluation for thrombectomy
Interventional procedure times		Door to needle time, door to inguinal puncture time
Thrombectomy		Date and time of inguinal puncture or stent implant or reperfusion; TICI^k^ scale, thrombolysis reperfusion

^a^PT: prothrombin time.

^b^aPTT: activated partial thromboplastin time.

^c^INR: international normalized ratio.

^d^NIHSS: National Institutes of Health Stroke Scale.

^e^TIA: transient ischemic attack.

^f^CT: computed tomography.

^g^CTA: computed tomography angiography.

^h^MRI=magnetic resonance imaging.

^i^ASPECTS=Alberta Stroke Program Early CT Scan.

^j^See details for risk mitigation in Table 4.

^k^TICI: Thrombolysis in Cerebral Infarction.

**Table 4:** Output information in the telestroke system.

Module	Value suggested by the system
Diagnoses	Hemorrhagic stroke; acute, subacute, or chronic ischemic stroke; large vessel occlusions; TIA^a^; stroke mimic
Outcomes	Neurosurgery; hemorrhagic care; hospitalization or neurology (if reperfusion risk, chronic or subacute ischemic stroke, TIA); recovery room (if hemodynamic stable); ICU^b^, after thrombolysis and/or thrombectomy; ambulatory care (with secondary prevention using statins or anticoagulants)
Transfer	Transfer from PHS^c^ to IHS^d^ or AHS^e^, transfer from IHS to AHS

^a^TIA: transient ischemic attack.

^b^ICU: intensive care unit.

^c^PHS: primary health care setting.

^d^IHS: intermediate health care setting.

^e^AHS: advanced health care setting.

### Software Development and Validation

The algorithms included in the three health care settings were incorporated into web-based software. Individual user profiles were created for the administrative staff and health care providers, who were assigned specific privileges.

A user-friendly interface reduces human error and assures the completeness and integrity of the information. The questionnaires implemented were straightforward and only required single-click selections instead of free-text typing for easy and rapid data input. The software was developed using the Hypertext Preprocessor and JavaScript languages and could be executed in any web browser on a laptop, tablet, or smartphone.

For data storage, a MySQL 5.1.40 database (Oracle Corporation, Redwood City, CA) was used, wherein sensitive data were encrypted (ie, patient identification). Data were stored in a structured relational database, allowing future evaluation of the system performance as well as a strong foundation for public health policies. The database included the administrative information of each facility in the telestroke network and the possible referral facilities (ie, those that have a given facility that was contracted to receive patients when a transfer is required). This information allowed the rapid selection of the most suitable stroke center according to the patient’s needs after a judicious assessment of the clinical requirements and transfer times. Given that “time is brain,” potential administrative pitfalls between primary health care settings and advanced health care settings also had to be considered for a quick and effective transfer. In our country, patients may be transported to various emergency departments until they are accepted in one of them, producing a critical delay in the required care known as “the death ride.”

Since a patient can arrive at any given hospital and may be transferred across several health care settings without receiving adequate stroke care, a “case” starting point was defined as the time when the first medical contact was documented in the last visited hospital until the final patient outcome was reported before the patient’s discharge. Therefore, when a patient is transferred to a second health care setting, all the information for the case is available in the receiving facility given that all data are stored in a server database and shared with all the facilities. This design decision allows common access to the patient’s health condition at any moment from any health care setting while also avoiding the entry of redundant information. Hence, past medical history, current clinical condition, blood test results, imaging evaluations, and procedures are available in real-time for all health care facilities across the whole spectrum of stroke patient care. In addition, this allows transfer reporting to the referral facilities ahead of the patient’s arrival, avoiding prolonged waiting times at emergency departments.

Software validation was performed in different phases. The first phase consisted of a simulation of the test scripts on all possible workflow pathways for each of the health care settings, which was performed to validate adequate software representation of each the algorithms. To validate the software implementation, more than 1211 test scripts were performed covering all possible pathways in each of the predefined health care settings. The second phase consisted of a retrospective registry of cases from our stroke database (nearly 600 patients in the last 5 years). The third phase was the validation of the software by neurology residents, who utilized the software while also performing a usual clinical assessment with printed forms. The final phase is to be performed between different health care facilities to test the performance of our telestroke network with real-life cases and transfers based on the information broadcast. The total number of possible pathways documented after this initial experience was as follows: 10 in the primary health care setting, 39 in the intermediate health care setting, and 1162 in the advanced health care setting.

The final system was named Telestroke-RU (copyright 13-70-240, 03/12/2018 from the National Copyright Office, Colombia) and is available for authorized users [28]. This system is not a product intended for commercial or profit uses and may be used for educational purposes.

## Discussion

### Principal Findings

The comprehensive telestroke platform developed in this work is the first stage to optimizing health care delivery for patients with stroke symptoms regardless of the entry point into our local emergency network in both urban and rural regions.

This system supports health care personnel by providing adequate stroke care and facilitating the prompt transfer of patients to a more appropriate health care setting according to the specific cerebrovascular disease at presentation. This system facilitates stroke care delivery for patients with acute ischemic stroke within the therapeutic window who are candidates for reperfusion therapies. Therefore, the system will contribute to mitigating the well-known mortality and morbidity associated with stroke.

Further evaluations will be performed to assess the true impact of this tool in terms of reductions in critical time windows, such as the time between symptom onset and reperfusion, door to needle time, primary health care setting to advanced health care setting transfer times, discharge clinical outcomes, accuracy of the final diagnosis, and the clinical outcomes of patients at 30 and 90 days using the modified Rankin scale [29].

### Comparison With Prior Work

To the best of our knowledge, in our country, there are no software tools for the assessment and management of patients with stroke symptoms. Worldwide, smartphone apps and web-based tools are available [30-34]. These solutions were designed for acute ischemic stroke care, for triage protocols, and as an aid for transfers when reperfusion therapies are needed (eg, Field Assessment Stroke Triage for Emergency Destination score) [30] as well as the delivery of efficient inpatient or ambulatory stroke care [32,34]. Other tools have been developed with the purpose of the evaluation of specific clinical scales or radiological scores [33]; these tools use the same algorithm independent of the level of resources available at the entry point to the health care system. In contrast, our system integrates 12 clinical and radiological scales, scores, and predictors according to the specific health care setting or the referral facility to provide a specific diagnosis (hemorrhagic stroke; acute, subacute, or chronic ischemic stroke; TIA; stroke mimic). Our system allows either immediate treatment or further transfer to the appropriate health care setting. Since our solution is not integrated with the hospital electronic health record (EHR) system, it can be used in all health care services, independent of the EHR system used at each facility. It is worth mentioning that, in our country, EHR systems are not available at several rural primary health care settings.

### Limitations

The continuous improvement of evidence-based stroke care guidelines motivates the continuous review of health care setting algorithms and, therefore, software updates. Further work includes the use of GPS and traffic applications to calculate the actual duration of real-time patient transfers and adequate selection of the referral facility with the shorter transfer time. In the short-term, this system will be migrated to a smartphone app to allow for a greater number of system users in a friendlier interface.

### Conclusions

The implementation of this system in a telestroke network contributes to the fulfillment of and adherence to recently published stroke care guidelines, providing evidence-based practice, improving patient outcomes, and supporting the achievement of several requirements to achieve and maintain primary stroke center certification.

This telestroke system allows the assessment of different therapeutic alternatives according to the specific patient’s clinical condition, thus improving efficiency and providing high-quality delivery of care. Finally, the epidemiological information stored in the database will inform public health care policies to design and implement better national policies for remote regions with significant underreporting of acute cerebrovascular diseases.

## Abbreviations

AHS: advanced health care setting.
aPTT: activated partial thromboplastin time.
ASPECTS: Alberta Stroke Program Early Computed Tomography Scan.
CT: computed tomography.
CTA: computed tomography angiography.
CTP: computed tomography perfusion.
DWI: diffusion-weighted magnetic resonance imaging.
EHR: electronic health record.
FAST-ED: Field Assessment Stroke Triage for Emergency Destination.
ICH: intracerebral hemorrhage.
ICU: intensive care unit.
IHS: intermediate health care setting.
INR: international normalized ratio.
IV rtPA: intravenous recombinant tissue plasminogen activator.
MRA: magnetic resonance angiography.
MRI: magnetic resonance imaging.
NIHSS: National Institutes of Health Stroke Scale.
PHS: primary health care setting.
PT: prothrombin time.
PTT: partial thromboplastin time.
TIA: transient ischemic attack.
TICI: Thrombolysis in Cerebral Infarction.
WFNS: World Federation of Neurosurgical Societies.
